# Tuning the Polarity of MoTe_2_ FETs by Varying the Channel Thickness for Gas-Sensing Applications

**DOI:** 10.3390/s19112551

**Published:** 2019-06-04

**Authors:** Asha Rani, Kyle DiCamillo, Md Ashfaque Hossain Khan, Makarand Paranjape, Mona E. Zaghloul

**Affiliations:** 1 School of Engineering and Applied Science, The George Washington University, Washington, DC 20052, USA; zaghloul@gwu.edu; 2 Department of Physics, Georgetown University, Washington, DC 20057, USA; Mak.Paranjape@georgetown.edu; 3 Department of Electrical and Computer Engineering, George Mason University, Fairfax, VA 22030, USA; mkhan53@gmu.edu

**Keywords:** 2D materials, MoTe_2_, channel thickness effect, polarity switching

## Abstract

In this study, electrical characteristics of MoTe_2_ field-effect transistors (FETs) are investigated as a function of channel thickness. The conductivity type in FETs, fabricated from exfoliated MoTe_2_ crystals, switched from p-type to ambipolar to n-type conduction with increasing MoTe_2_ channel thickness from 10.6 nm to 56.7 nm. This change in flake-thickness-dependent conducting behavior of MoTe_2_ FETs can be attributed to modulation of the Schottky barrier height and related bandgap alignment. Change in polarity as a function of channel thickness variation is also used for ammonia (NH_3_) sensing, which confirms the p- and n-type behavior of MoTe_2_ devices.

## 1. Introduction

Since the discovery of graphene in 2004 [1], the two-dimensional (2D) layered materials have attracted significant attention for device applications owing to their unique physical properties and promising applications in nanoelectronic devices and circuits [1,2,3,4,5]. Due to lack of bandgap structure, graphene-based transistors are difficult to switch off, which is critical for electronic devices [6]. This limitation in graphene led researchers towards layered semiconductor transition metal dichalcogenides (TMDs), which are now seen as promising candidates for next-generation transistors due to their large variety of bandgap values [7,8], high charge-carrier mobility [9,10], and high on/off current ratio (~10^6^) [11]. Transition metal dichalcogenide (TMDC) compounds consists of one layer of transition-metal sheet (Mo and W) sandwiched between two sheets of chalcogenide elements (S, Se, and Te). Weak van der Waals interactions between the layers facilitate exfoliation of TMDCs crystals down to a single layer [3].

Among all TMDCs, MoTe_2_ is the only material which can be grown in two phases: semiconducting (2H-phase) and metallic (1T’-phase). The theoretical bandgap for bulk and single-layer MoTe_2_ in the semiconducting phase (2H) is 0.81 eV (indirect) and 1.13 eV (direct), respectively [12,13,14,15]. The bandgap of MoTe_2_ is close to that of Si (1.1eV), making it an attractive candidate for optoelectronic devices with a range spreading from visible to near-infrared [1,11,16]. Recent literatures show that p- and n-type carrier injection can be obtained by work-function engineering of the contact metals [17]. The high work function of Pt and low work function of Ti metal contact can strengthen the p- and n-type conducting behavior [17]. It has been demonstrated that the field-effect transistor (FET) polarity can be electrostatically altered by dual top gate geometry [18]. The ambipolar behavior of MoTe_2_ changing to n-type upon exposure to UV light for 2 h has also been reported [19]. It was also observed that unipolar p-type behavior of MoTe_2_ flakes with Ti/Au metal contact and environmental oxygen can tune the device from ambipolar to unipolar p-type [11]. 2D layered nanomaterials are advantageous for gas-sensing applications due to their high surface-to-volume ratio, which facilitates surface reactions [20,21,22,23]. For chemiresistive-type FET gas sensors, electrical resistivity or conductivity are altered upon adsorption of target molecules on the surface of 2D nanomaterials [19,24,25]. It is a well-known fact that NH_3_ acts as an electron donor (n-type doping), resulting in an increase of resistance for p-type semiconductors based on the charge-transfer mechanism [26].

In this study, we demonstrate that the change in polarity of MoTe_2_ is a function of channel thickness. From our measurements, we observed that MoTe_2_ FETs showed p-type, ambipolar, and n-type polarity with increasing flake thickness. MoTe_2_ is intrinsically p-doped, but can also exhibit an ambipolar behavior [27]. To date, no clear evidence about unipolar n-type behavior of MoTe_2_ FETs has been reported. From our experimental results of thin (~5.6–12 nm), medium (~12–45 nm), and thick (>45 nm) channel devices from tellurium tetrachloride (TeCl_4_)-based transport-agent devices showed, p-, ambipolar-, and n-type conducting behavior, respectively. In this study we also highlighted the effect of channel thickness on gas-sensing application of MoTe_2_ devices, which has not been mentioned in early literature [19]. The NH_3_ gas-sensing results of MoTe_2_ FETs further confirms the change in polarity as a function of flake thickness. No additional fabrication steps are required to obtain unipolar p- or n-type FETs. This electrical property of MoTe_2_ can provide guidance to obtain p-type, ambipolar, and n-type devices, merely by tuning the channel thickness.

## 2. Materials and Methods

**Figure 1:**
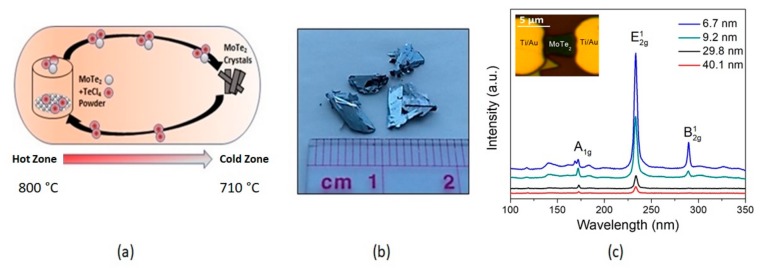
(**a**) Schematic diagram of the two-zone electric furnace used for the growth of 2H-MoTe_2_ crystal using TeCl_4_ as transport agent; (**b**) As-synthesized bulk of MoTe_2_ crystals after growth; (**c**) Raman spectra of MoTe_2_ flakes of different thickness exfoliated from bulk MoTe_2_ single crystals grown by chemical vapor transport (CVT) with TeCl_4_ transport agent. Inset shows plane-view optical image of FET device with a 4.6 nm thick channel.

In this work, MoTe_2_ flakes were exfoliated from bulk crystals grown by a chemical vapor transport (CVT) method. MoTe_2_ (2H-phase) crystal was prepared by mixing powders of MoTe_2_ and a small amount (ca 5 mg/cm^3^) of transport agent (TeCl_4_), vacuum sealed in quartz ampoule for 140 h, and placed inside a furnace with temperature gradient. The temperature of MoTe_2_ charge was maintained at 800 °C (hot zone), and the opposite end of ampoule was at 710 °C (cold zone). The ampoule was slowly cooled after seven days of growth [28]. The schematic diagram of the setup used for MoTe_2_ crystal growth and an image of bulk crystal after CVT growth is shown in Figure 1a,b respectively. This method is known to produce pure 2H-phase of MoTe_2,_ as verified by X-ray powder diffraction (XRD), Transmission Electron Microscopy (TEM) [29], and Raman spectroscopy (Figure 1c).

Different thickness of flakes ranging from 5.6 nm to 60 nm were obtained from CVT-grown bulk MoTe_2_ crystal, and transferred onto SiO_2_/Si substrate by mechanical exfoliation (Supplementary Figure S1). The thickness of the SiO_2_ layer was 285 nm. The blank SiO_2_/Si substrate was patterned with design having numbers, symbols, and alphabets using photolithography. For fabrication steps involving photolithography, a bi-layer stack of positive photoresists LOR 3A and Microposit SPR 220.3 was used. LOR 3A was spin-coated at 4000 revolutions per minute (rpm), followed by a soft bake at 115 °C for 2 min. Next, Microposit SPR 220.3 was spin coated at 4000 rpm for 45 s and soft baked at 115 °C for 2 min. The samples were then exposed to UV illumination using mask-aligner (MA6 SUSS Microtec) and developed in Microposit CD 26A for 30 s, followed by a rinse in deionized water. Metal deposition in all fabrication steps was performed using an e-beam evaporator (Denton Infinity 22), and a lift-off process was performed by immersing the devices in remover 1165 at 80 °C for 30 min. These pattern marks were used for locating the desired MoTe_2_ flakes after mechanical exfoliation. Prior to exfoliation, the patterned substrate was ultrasonically cleaned in acetone, iso-propanol (IPA), and deionized water (DIW), followed by oxygen plasma cleaning to remove the adsorbates from the surface [30]. Under the Olympus optical microscope, successful transfer of few layers of MoTe_2_ flakes on SiO_2_/Si substrate was mapped out to pre-fabricated marking points for a source/drain contact pattern. Fabrication steps shown in Supplementary Figure S2, was also used for patterning the source/drain metal contact of MoTe_2_ devices. After fabrication, the devices were thermally annealed in a vacuum environment at 350 °C for 5 min to reduce the contact resistance between metal and MoTe_2_ flake. Further, atomic force microscopy (AFM) was used to confirm the exact channel thickness and verify uniformity, absence of folds and cracks on the MoTe_2_ flake under investigation. 

**Figure 2:**
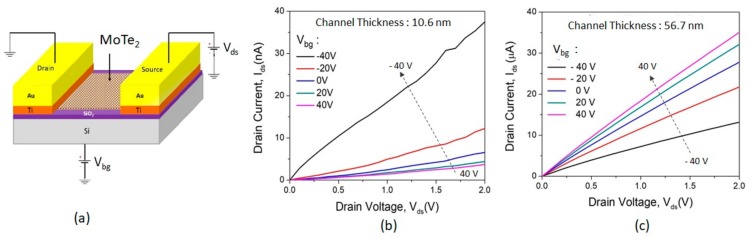
(**a**) Schematic of back-gate field-effect transistor (FET) connection. Output characteristics of MoTe_2_ FETs under different back-gate voltages, V_bg_. FETs with channel thickness of (**b**) 10.6 nm and (**c**) 56.7 nm showing p- and n-type conductivity, respectively.

After exfoliation, back-gate FET measurement was done using a Lakeshore probe station. The schematic of the back-gate FET connection is shown in Figure 2a. The silicon of substrate was used as a back-gate electrode and the SiO_2_ layer acted as the gate dielectric in the back-gate FET measurement. These devices were characterized by output (Figure 2b) and transfer (Figure 2c) curves using back-gate FET measurement at room temperature. After the back-gate FET measurement, the devices were verified by NH_3_ gas sensing for n- and p-type conducting behavior for the thick and thin channel. The gas sensing was performed by exposing 100 ppm of NH_3_ concentration diluted with N_2_ as carrying gas. N_2_ gas is used to dilute NH_3_ to the desired concentration at room temperature. We used N_2_ as diluting gas due to its better recovery when compared with air environment. The gas sensing performance of the device was evaluated by applying 5 V between source and drain, and ground to the back-gate electrode. 

## 3. Results

The flake-thickness scaling effect on MoTe_2_ so far has gained less attention for the use in electronics compared with their thin counterparts. In this study, we have examined the conducting behavior of MoTe_2_ FETs prepared using a TeCl_4_ transport agent by CVT method. Using Raman spectroscopy with 532 nm laser source, we identified the lattice vibrational modes of mechanically exfoliated 2H-MoTe_2_ flakes. The Raman spectra in Figure 1c exhibits characteristic A_1g_ at 170 cm^−1^, E^1^_2g_ at 235 cm^−1^, and B_2g_ at ≈288 cm^−1^ modes (the latter is active only in thin layers), which is in good agreement with studies reported in the literatures [31]. This verifies the 2H-phase of MoTe_2_ and good crystalline quality used in FETs. Inset in Figure 1c shows the optical image of 4.6 nm fabricated MoTe_2_ FET. Optical imaging technique has been popular in the rough estimate of flake thickness [32].

To understand the channel-thickness effect of FET transport characteristics, we measured the output (drain current (I_ds_) vs. drain voltage, (V_ds_)) and transfer (I_ds_ vs. back-gate voltage (V_bg_)) characteristics using Lakeshore probe station. The schematic diagram of circuit connection for back-gate FET measurement is shown in Figure 2a. In Figure 2b, the output characteristics of the 10.6 nm MoTe_2_ FET shows an increase in conduction as gate voltage decreases from +40 V to –40 V, showing that the majority of carriers are holes, since the channel is entering the ON state with a negative gate voltage. Contrarily, the output characteristic of the 56.7 nm MoTe_2_ device, shown in Figure 2c, shows that the majority of carriers in the channel are electrons (ON state with positive gate voltage) as gate voltage decreases from +40 V to –40 V. Clearly, p-type and n-type transport behavior is observed for 10.6 nm vs. 56.7 nm MoTe_2_ FETs, respectively. The nonlinear behavior of the output curve in Figure 2b,c can be attributed to a small Schottky barrier at the metal/semiconductor junction due to difference between the work function of Ti (4.33 eV) and the electron affinity of MoTe_2_ (4.3 ± 0.1 eV) [17,33].

**Figure 3:**
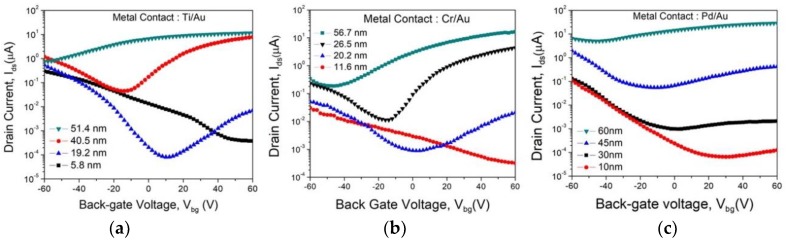
Transfer characteristics at V_ds_ = 2V of TeCl_4_-grown and MoTe_2_ FETs with different channel thickness for (**a**) Ti/Au; (**b**) Cr/Au, and (**c**) Pd/Au.

We further studied the transfer behavior of the FETs at V_ds_ = 2 V. The transfer characteristic results showed that thick, medium, and thin channel are n-type, ambipolar, and p-type, respectively. The gate leakage current (I_gs_) measured in the pA range (negligible). The maximum on/off current ratio obtained was ~1 × 10^4^, which increases with decreasing channel thickness. Figure 3 shows transfer curves for varying channel thickness for different metal contacts. Multiple devices with various metal contact (Ti/Au, Cr/Au, and Pd/Au), were fabricated which reproduced similar transfer characteristic as a function of MoTe_2_ channel thickness [34]. The metal work functions (ϕ_m_) of Ti, Cr, and Pd are 4.3 eV, 4.8 eV, and 5.1 eV, respectively. At least 10 devices from each metal type were fabricated and tested to reproduce the transfer curves to verify the change in polarity based on channel thickness. From transfer curves, we observed that MoTe_2_ FETs showed p-type behavior for very thin channels, ≈5–15 nm and ambipolar behavior for medium channel thicknesses, from ≈15 to 50 nm. For channels thicker than ≈50 nm, FETs showed n-type unipolar behavior. Previous studies on TMDC (such as WSe_2_, MoS_2,_ and MoTe_2_) have presented the effect of channel thickness on various transport properties in FETs [35,36,37,38] , but a wide range of channel thickness (~5–60 nm) and its effect on polarity of MoTe_2_ devices has not been highlighted until now. 

In reference to n-type conducting behavior, the n-type doping has been observed in TMDCs when TeCl_4_ is used as the transport agent in CVT growth [39] and during post-growth chloride molecular doping of TMDC compounds [40]. In this work, we assume that the Cl doping is playing a dominant role for n-type behavior in thick channel devices. In thin-channel FETs, conductivity switches to p-type, indicating the diminishing of Cl doping. We attribute it to the increase in surface defects and adsorbates in ultra-thin layers, which is also exemplified in atomically thin MoS_2_ FETs [35]. In addition to the possible effect of reduced MoTe_2_ channel thickness on n-type doping efficiency, we also speculate that the polarity switching from n- to p-type in thinner layers may be caused by the modulation of Schottky barrier height (SBH) and corresponding band alignment and band-bending at the metal/MoTe_2_ interface. The effective barrier height for Ti, Cr, and Pd contacts are 41.1, 40.3, and 10.2 meV, respectively [41,42].

**Figure 4:**
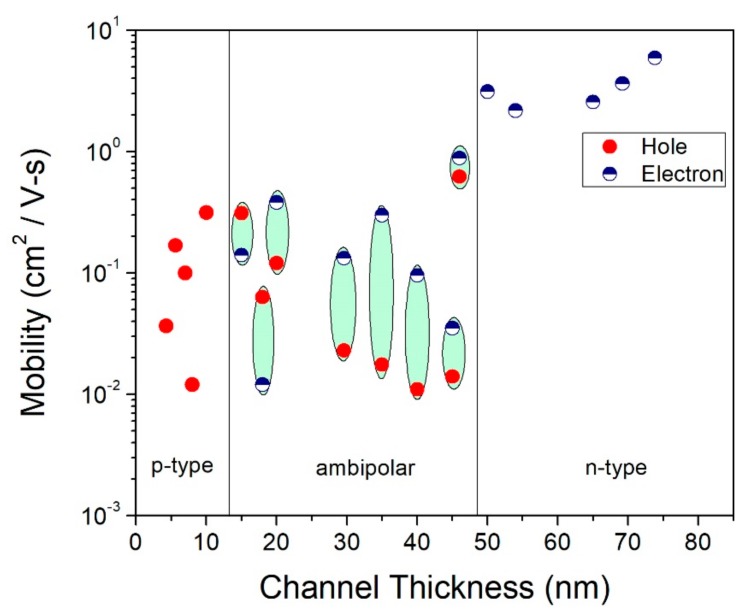
Field-effect mobility of electrons and holes vs. channel thickness for MoTe_2_ FETs. Encircled pairs of data points correspond to the ambipolar devices that exhibit ambipolar conductivity.

We also investigated the field-effect carrier mobility (µ_FE_) of fabricated devices extracted from the transfer curve of Figure 3a and MoTe_2_ devices with Ti/Au as metal contact. Mobility, µ_FE_ of the back-gated MoTe_2_ FETs is defined as follows:

µ_FE_ = (dI_ds_/dV_bg_) {L/(W C_OX_ V_ds_)} (1)

where C_OX_ (=ε_0_·ε_r_/d) is silicon oxide capacitance per unit area, d is the oxide thickness, ε_0_ and ε_r_ are the relative permittivity of free space and the relative permittivity of SiO_2,_ respectively, L and W are the channel length and width, respectively, V_ds_ is the drain-source voltage, and (dI_ds_/dV_bg_) is the inverse slope of transfer characteristic in the linear region of the ON state. The mobility increases with increasing channel thickness, as shown in Figure 4. A thicker channel has higher mobility compared with thinner MoTe_2_ devices. The influence of channel thickness in MoTe_2_ devices can be associated to Coulomb scattering and quantum confinement, as reported in early literatures [43,44]. Coulomb interactions weaken the scattering of carriers, resulting in higher mobility for thicker FETs compared with thinner counterparts, which was also demonstrated for both MoTe_2_ [35] and MoS_2_ [45] FETs.

**Figure 5:**
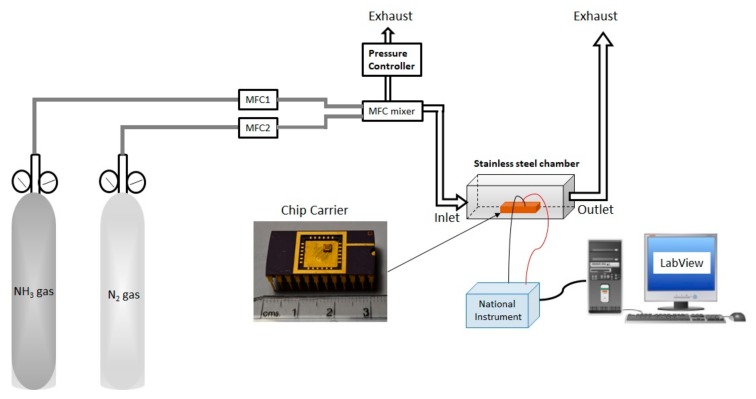
Schematic of gas-sensing setup.

To further confirm the change in polarity of FET devices, their response in the presence of gas molecules was measured. Figure 5 shows the gas-sensing setup used for NH_3_ sensing in this work. Nitrogen (N_2_) as diluting gas is connected to MFC2 and mixed with NH_3_ target gas (connected to MFC1). Both MFC1 and MFC2 are mixed in MFC mixer for the required concentration. The output of MFC1 and MFC2 is connected to MFC mixer for the required concentration of NH_3_ to flow in the gas-sensing stainless steel chamber for measurement. The MFC mixer is also connected to a pressure controller used to vent out extra gases. The MoTe_2_ device is packaged on ceramic chip carrier (inlet of Figure 5) and placed inside the stainless steel chamber, and a source/drain is connected to probes of a National Instrument (NI) for data collection. The gas sensing data from the NI instrument is analyzed using LabView program by transient curve. 

**Figure 6:**
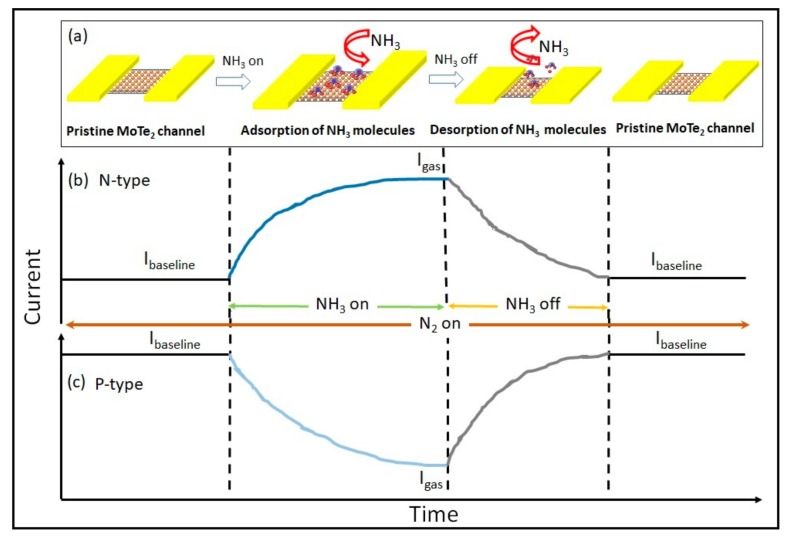
(**a**) Mechanism of NH_3_ sensing. Schematic showing current–time curve of NH_3_ sensing by (**b**) n-type and (**c**) p-type MoTe_2_ surface.

The schematic of NH_3_ sensing on n- and p-type is shown in Figure 6a–c. Figure 6a shows the schematic of process flow for NH_3_ gas sensing. We selected NH_3_ as the target gas since it is a strong electron donor and will produce opposite behavior in n-type (Figure 6b) and p-type (Figure 6c) devices. When NH_3_ is adsorbed on the n-type MoTe_2_ (Figure 6b) surface, an increase in current is observed due increase in electron charge carriers on the MoTe_2_ surface. When NH_3_ gas is switched off, NH_3_ molecules are desorbed from the surface and the current value of pristine-state MoTe_2_ surface is obtained. Similarly, when NH_3_ is adsorbed on the p-type MoTe_2_ surface, a decrease in current value is seen, due to a decrease in hole-charge carriers on the MoTe_2_ surface, as shown in Figure 6b. A single pulse of 100 ppm concentration of NH_3_ was introduced to the MoTe_2_ devices, and the current response of devices was measured.

**Figure 7:**
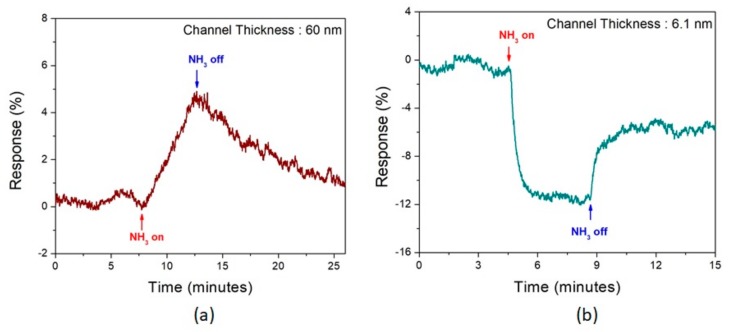
Response to 100 ppm NH_3_ in air of (**a**) 60 nm and (**b**) 6.1 nm thick MoTe_2_ devices, confirming n- and p-type conductivity, respectively. Note much faster recovery time for the thin flakes.

Figure 7a,b shows the gas-sensing response of 100 ppm NH_3_ of 60 nm and 6.1 nm MoTe_2_ flakes. We were able to detect the NH_3_ concentration as low as 1 ppm for thin MoTe_2_ channels. We couldn’t go below 1 ppm due to a limitation of the mass-flow controller. The lowest concentration detected by thicker channel devices was 100 ppm. Hence, for comparison purpose of n- and p-type MoTe_2_ devices, 100 ppm NH_3_ sensing was used in this work. The thick (60 nm), n-type MoTe_2_ FET showed an expected increase in current, and the thin, p-type MoTe_2_ FET showed a corresponding decrease in current. The response is calculated using Equation (2) for Figure 7.

Response (%) = [(I_gas_ − I_baseline_)/I_baseline_] × 100 (2)

where, I_gas_ and I_baseline_ are currents when NH_3_ is on and the baseline current, as shown in Figure 6b,c. This experiment verifies the change in polarity in MoTe_2_ flakes due to variation in channel thickness. Thin flakes show higher sensitivity (~12 %) compared with thick flakes (~5%) due to high surface-to-volume ratio. The current values of NH_3_ sensing by thin and thick MoTe_2_ flakes is shown in Supplementary Figure S3. The low value of sensitivity and baseline drift in devices can be due to the limitations of the custom-built gas-sensing setup. In this work, we showed that electrical properties of MoTe_2_ are dependent on flake thickness, which is also verified by NH_3_ sensing of thin and thick flakes. No extra fabrication steps are required to obtain the n- or p-type conduction behavior in MoTe_2_ FETs. We predict that with further experiments and modifications in our gas-sensing setup, a similar change in polarity of MoTe_2_ device as function of channel thickness can also be shown for NH_3_ sensing in an air environment. 

## 4. Conclusions

In this work, we demonstrated a simple and effective way to fabricate p-type, ambipolar, and n-type MoTe_2_ FETs just by tuning the channel thickness. FETs were fabricated from mechanically exfoliated flake from CVT-grown single-crystal 2H-MoTe_2_. The electrical properties of devices prepared from 2H-MoTe_2_ flakes were examined for varying thickness (~5–60 nm) using the output and transfer curves. FETs showed polarity switching from n-type through ambipolar to p-type with decreasing channel thickness from 60 nm to ~5.6 nm. The n-type transfer behavior in thick-channel MoTe_2_ FET is attributed to chlorine doping from the TeCl_4_ transport agent used in CVT growth of bulk crystals. The switching of polarity by thinning the FET channel may be associated with the increasing role of surface states in ultra-thin layers, which can influence charge-carrier concentration by modulating the Schottky barrier height between metal and semiconductor interface. This study also showed the NH_3_ gas-sensing application of p- and n-type MoTe_2_ devices, indicating the change in polarity due to channel thickness. 

## Supplementary Materials

The following are available online at https://www.mdpi.com/1424-8220/19/11/2551/s1, Figure S1, Process steps for plasma–assisted mechanical exfoliation; Figure S2, Process steps showing fabrication of source/drain contacts on exfoliated samples; Figure S3, Transient curve (current-time) of (a) 60 nm and (b) 6.1 nm MoTe_2_ flakes for NH_3_ sensing.

## References

[1] Radisavljevic B., Radenovic A., Brivio J., Giacometti V., Kis A. (2011). Single-layer MoS_2_ Transistors. Nat. Nanotechnol..

[2] Kim K.S., Zhao Y., Jang H., Lee S.Y., Kim J.M., Kim K.S., Ahn J.-H., Kim P., Choi J.-Y., Hong B.H. (2009). Large-scale pattern growth of graphene films for stretchable transparent electrodes. Nature.

[3] Novoselov K.S., Geim A.K., Morozov S.V., Jiang D., Zhang Y., Dubonos S.V., Grigorieva I.V., Firsov A.A. (2004). Electric field effect in atomically thin carbon films. Science.

[4] Lopez-Sanchez O., Lembke D., Kayci M., Radenovic A., Kis A. (2013). Ultrasensitive photodetectors based on monolayer MoS2. Nat. Nanotechnol..

[5] Yin Z., Li H., Jiang L., Shi Y., Sun Y., Lu G., Zhang Q., Chen X., Zhang H. (2012). Single-layer MoS2 phototransistors. ACS Nano.

[6] Meric L., Han M.Y., Young A.F., Ozyilmaz B., Kim P., Shepard K.L. (2008). Current saturation in zero-bandgap, top-gated graphene field-effect transistors. Nat. Nanotechnol..

[7] Tang Q., Zhou Z. (2013). Graphene-analogous low-dimensional materials. Prog. Mater. Sci..

[8] Fiori G., Bonaccorso F., Iannaccone G., Palacios T., Neumaier D., Seabaugh A., Banerjee S.K., Colombo L. (2014). Electronics based on two-dimensional materials. Nat. Nanotechnol..

[9] Strait J.H., Nene P., Rana F. (2014). High Intrinsic Mobility and Ultrafast Carrier Dynamics in Multilayer Metal-Dichalcogenide MoS_2_. Phys. Rev. B.

[10] Bao W.Z., Cai X.H., Kim D., Sridhara K., Fuhrer M.S. (2013). High Intrinsic Mobility and Ultrafast Carrier Ambipolar MoS_2_ Field-Effect Transistors: Substrate and Dielectric Effects. Appl. Phys. Lett..

[11] Pradhan N.R., Rhodes D., Feng S.M., Xin Y., Memaran S., Moon B.-H., Terrones H., Terrones M., Balica L. (2014). Field- Effect Transistors Based on Few- Layered alpha-MoTe_2_. ACS Nano.

[12] Ding Y., Wang Y., Ni J., Shi L., Shi S., Tang W. (2011). First principles study of structural, vibrational and electronic properties of graphene-like MX2 (M = Mo, Nb, W, Ta; X = S, Se, Te) monolayers. Phys. B.

[13] Ataca C., Sahin H., Ciraci S. (2012). Stable, Single-Layer MX_2_ Transition-Metal Oxides and Dichalcogenides in a Honeycomb-Like Structure. J. Phys. Chem. C.

[14] Ma Y., Dai Y., Guo M., Niu C., Lu J., Huang B. (2011). Electronic and magnetic properties of perfect, vacancy-doped, and nonmetal adsorbed MoSe_2_, MoTe_2_ and WS_2_ monolayers. Phys. Chem. Chem. Phys..

[15] Kuiri M., Chakraborty B., Paul A., Das S., Sood A.K., Das A. (2016). Enhancing photoresponsivity using MoTe_2_-graphene vertical heterostructures. Appl. Phys. Lett..

[16] Li H.-M., Lee D.-Y., Choi M.S., Qu D., Liu X., Ra C.-H., Yoo W.J. (2014). Metal-Semiconductor Barrier Modulation for High Photoresponse in Transition Metal Dichalcogenide Field Effect Transistors. Sci. Rep..

[17] Nakaharai S., Yamamoto M., Ueno K., Tsukagoshi K. (2016). Carrier Polarity Control in α-MoTe_2_ Schottky Junctions Based on Weak Fermi-Level Pinning. ACS Appl. Mater. Interfaces.

[18] Resta G.V., Sutar S., Balaji Y., Lin D., Raghavn P., Radu I., Catthoor F., Thean A., Gaillardon P.-E., Micheli G. (2016). Polarity control in WSe_2_ double-gate transistors. Sci. Rep..

[19] Feng Z., Xie Y., Wu E., Yu Y., Zheng S., Zhang R., Chen X., Sun C., Zhang H., Liu J. (2017). Enhanced Sensitivity of MoTe_2_ Chemical Sensor through Light Illumination. Micromachines.

[20] Ping J., Fan Z., Sindoro M., Ying Y., Zhang H. (2017). Recent advances in sensing applications of two-dimensional transition metal dichalcogenide nanosheets and their composits. Adv. Funct. Mater..

[21] Yang S., Jiang C., Wei S.-H. (2017). Gas sensing in 2D materials. Appl. Phys. Rev..

[22] Choi S.J., Kim I.D. (2018). Recent developments in 2D Nanomaterials for Chemiresistive-Type Gas Sensors. Electron. Mater. Lett..

[23] Liu X., Ma T., Pinna N., Zhang J. (2017). Two-dimensional layered nanostructured materials for gas-sensing. Adv. Funct. Mater..

[24] Cho B., Hahm M.G., Choi M., Yoon J., Kim A.R., Lee Y.-L., Park S.-G., Kwon J.-D., Kim C.S., Song M. (2015). Charge-transfer based gas sensing using atomic-layer MoS_2_. Sci. Rep..

[25] Liu B., Chen L., Liu G., Abbas A.N., Fathi M., Zhou C. (2014). High-performance chemical sensing using Schottky-contacted chemical. ACS Nano.

[26] Late D.J., Huang Y.-K., Liu B., Acharya J., Shirodkar S.N., Luo J., Yan A., Charles D., Waghmare U.V., Dravid V.P. (2013). Sensing behavior of atomically thin-layered MoS_2_ transistors. ACS Nano.

[27] Lezama I.G., Ubaldini A., Longobardi M., Giannini E., Renner C., Kuzmenko A.B., Morpurgo A.F. (2015). Indirect-to-direct band-gap crossover in few-layer MoTe_2_. Nano Lett..

[28] Singh-Miller N.E., Marzari N.N. (2009). Surface energies, work functions, and surface relaxations of low index metallic surfaces from first principles. Phys. Rev. B. Condens. Matter.

[29] Oliver S.M., Beams R., Krylyuk S., Kalish I., Singh A.K., Bruma A., Tavazza F., Joshi J., Stone I.R., Stranick S.J. (2017). The structural phases and vibrational properties of Mo_1−x_W_x_Te_2_ alloys. 2D Mater..

[30] Huang Y., Sutter E., Shi N.N., Zheng J., Yang T., Englund D., Gao H.-J., Sutter P. (2015). Reliable exfoliation of large-area high-quality flakes of graphene and other two–dimensional materials. ACS Nano.

[31] Benameur M.M., Radisavljevic B., Heron J.S., Sahoo S., Berger H., Kis A. (2011). Visibility of dichalcogenide nanolayers. Nanotechnology.

[32] Golasa K., Grzeszczyk M., Molas M.R., Zinkiewicz M., Bala L., Nogajewski K., Potemski M., Wysmolek A., Babinski A. (2017). Resonant quenching of Raman scattering due to out-of-plane A_1g_/A’_1_ modes in few-layer MoTe_2_. Nanophotonics.

[33] Shimada T., Shuchi S.F., Parkinson A.B. (1994). Work function of photothreshold of layered metal dichalcogenides. Jpn. J. Appl. Phys..

[34] Rani A., DiCamillo K., Krylyuk S., Debnath R., Taheri P., Pranajape M., Korman C.E., Zaghloul M.E., Davydov A.V. Control of Polarity in Multilayer MoTe_2_ Field-Effect Transistors by Channel Thickness. Proceedings of the Low-Dimensional Materials and Devices 2018.

[35] Hyunjin J., Gwanmu L., Min-Kyu J., Yoojoo Y., Hojoon Y., Ji-Hoon P., Dongseok S., Chu S.L. (2017). Thickness-dependent carrier mobility of ambipolar MoTe_2_: Interplay between interface trap and Coulomb scattering. Appl. Phys. Lett..

[36] Perello J.D., Chae H.S., Song S., Lee H.Y. (2015). High-performance n-type black phosphorous transistors with type control via thickness and contact- metal engineering. Nat. Commun..

[37] Kwon J., Lee Y.J., Yu J.Y., Lee H.C., Cui X., Hone J., Lee H.G. (2017). Thickness-dependent Schottky barrier height of MoS_2_ field-effect transistors. Nanoscale.

[38] Cai Y., Zhang G., Zhang W.Y. (2014). Layer-dependent band alignment and work function of few-layer phosphorene. Sci. Rep..

[39] Legma J.B., Vacquier G., Casalot A. (1993). Chemical vapour transport of molybdenum and tungsten diselenides by various transport agents. J. Cryst. Growth.

[40] Yang L., Majumdar K., Liu H., Du Y., Wu H., Hatzistergos M., Hung P., Tieckelmann R., Tsai W., Hobbs C. (2014). Chloride Molecular Doping Technique on 2D Materials: WS_2_ and MoS_2_. Nano Lett..

[41] Townsend N.J., Amit I., Cracium M.F., Russo S. (2018). Sum 20 meV Schottky barriers in metal/MoTe_2_ Junctions. 2D Mater..

[42] Attena J.J., Uijttewaal M.A., Wijs G.A., Groot R.A. (2008). Work Function Anisotropy and surface stability of half-metallic CrO_2_. Phys. Rev. B.

[43] Liu Y., Stradins P., Huai S.H. (2016). Van der Waals metal-semiconductor junction: Weak Fermi level pinning enables effective tuning of Schottky barrier. Sci. Adv..

[44] Kim C., Moon I., Lee D., Choi M.S., Ahmed F., Nam S., Cho Y., Shin H.-J., Park S., Yoo W.J. (2017). Fermi level pinning at electrical metal contacts of monolayer molybdenum dichalcogenides. ACS Nano.

[45] Li S.-L., Wakabayashi K., Xu Y., Nakaharai S., Komatsu K., Li W.-W., Lin Y.-F., Aparecido-Ferreira A., Tsukagoshi K. (2013). Thickness-dependent interfacial Coulomb scattering in atomically thin field-effect transistors. Nano Lett..

