# Supplementary material for: Tuning the Polarity of MoTe2 FETs by Varying the Channel Thickness for Gas-Sensing Applications
Source: Sensors (Basel). 2019 Jun 4;19(11):2551. doi: 10.3390/s19112551 (PMC6603731; doi:10.3390/s19112551)
Supplement: Supplementary file 1 [file sensors-19-02551-s001.pdf]

Tuning the Polarity of $MoTe_2$ FETs by Varying the Channel Thickness for Gas Sensing Applications

Asha Rani [1,*], Kyle DiCamillo [2,*], Md Ashfaque Hossain Khan [3], Makarand Paranjape [2] and Mona E. Zaghloul [1]

[1] School of Engineering and Applied Science, The George Washington University, Washington, DC 20052, USA; zaghloul@gwu.edu

[2] Department of Physics, Georgetown University, Washington, DC 20057, USA; kd598@georgetown.edu; Mak.Paranjape@georgetown.edu

[3] Department of Electrical and Computer Engineering, George Mason University, Fairfax, VA 22030, USA; mkhan53@gmu.edu

* Correspondence: arani@gwmail.gwu.edu (A.R.); kd598@georgetown.edu (K.D.)

1. S1 Mechanical Exfoliation

$MoTe_2$ bulk flakes were transferred by mechanical exfoliation onto SiO_2/Si substrate. Figure S1 shows the process flow of exfoliation method used in this work. The substrate was sonicated in acetone, isopropanol and deionized water followed by O_2 plasma cleaning. The bulk $MoTe_2$ crystal was mechanically exfoliated using tape and transferred onto cleaned substrate and heated on laboratory hot plate at 80 ˚C with tape for two min. The tape is peeled from the substrate at room temperature completing the exfoliation process. Optical image of exfoliated substrate with target flake to fabricate device is shown in figure S1.

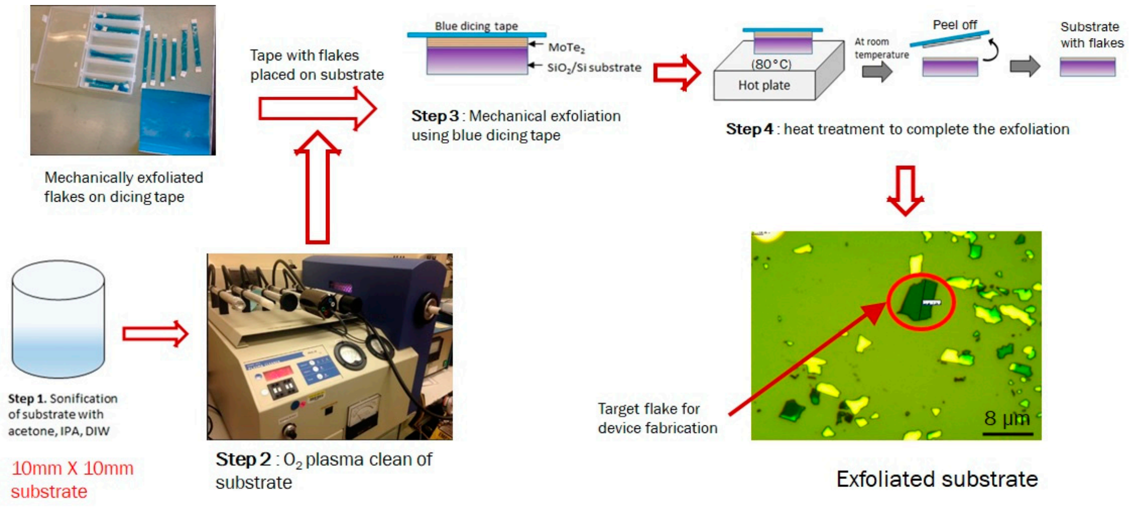

Figure S1. Process steps for plasma – assisted mechanical exfoliation.

2. S2 Device Fabrication

For fabrication steps involving photolithography, a bi-layer stack of positive photoresists LOR 3A and Microposit SPR 220.3 was used. LOR 3A was spin coated at 4000 revolutions per minute (rpm) followed by a soft bake at 115 °C for 2 min. Next Microposit SPR 220.3 was spin coated at 4000 rpm for 45 s and soft baked at 115 °C for 2 min. The samples were then expose to UV illumination using mask-aligner (MA6 SUSS Microtec) and developed in Microposit CD 26A for 30 s followed by rinse in DI water. Metal deposition in all fabrication steps was performed using e-beam evaporator (Denton Infinity 22) and lift-ff process was performed by immersing the devices in remover 1165 at 80 °C for 30 min.

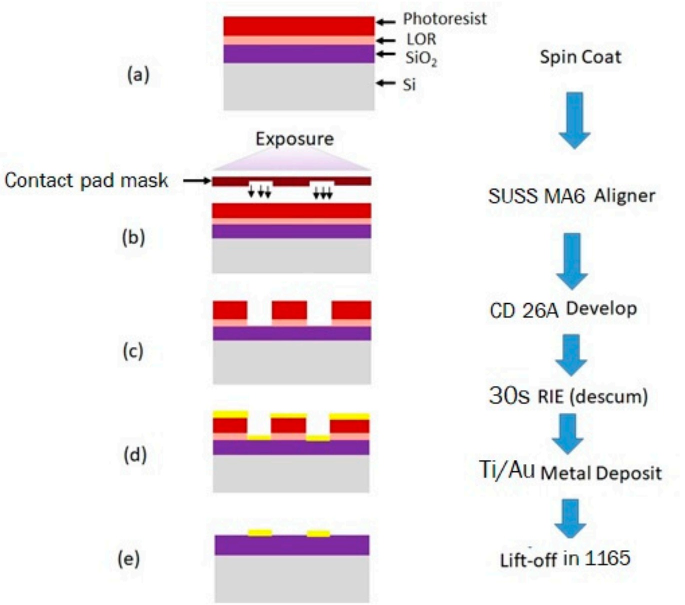

Figure S2. Process steps showing fabrication of source/drain contacts on exfoliated samples.

3. S3: Current-Time Transfer Curve of NH_3 Sensing

The transient curves of 60 nm (figure S3 (a)) and 6.1 nm (figure S3 (b)) $MoTe_2$ flake for NH_3 gas sensing is shown in figure S3. The thick flakes shows increase in current value when NH_3 molecules is adsorbed on the $MoTe_2$ surface indicating n-type polarity, whereas, thin flakes shows decrease in current value indicating p-type polarity of channel.

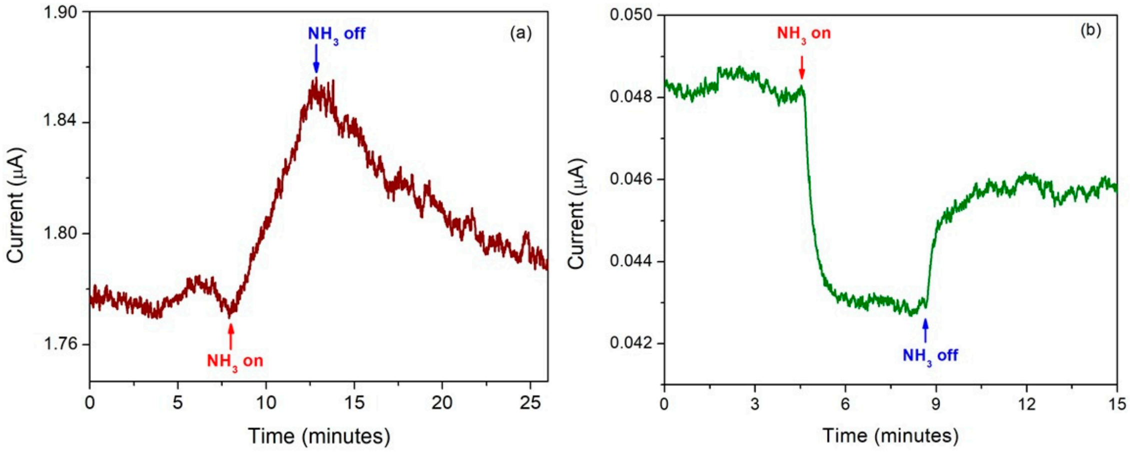

Figure S3. Transient curve (current-time) of (a) 60 nm and (b) 6.1 nm $MoTe_2$ flakes for NH_3 sensing.
